# Bilateral apical vertebral derotation technique by vertebral column manipulation compared with vertebral coplanar alignment technique in the correction of lenke type 1 idiopathic scoliosis

**DOI:** 10.1186/1471-2474-14-175

**Published:** 2013-05-31

**Authors:** Lin Sun, Yueming Song, Limin Liu, Yonggang An, Chunguang Zhou, Zhongjie Zhou

**Affiliations:** 1 Department of Orthopedics, West China Hospital, Sichuan University, Chendu 610041, China

**Keywords:** Idiopathic scoliosis, Three-dimensional correction, Bilateral apical vertebral derotation, Vertebral column manipulation, Vertebral coplanar alignment

## Abstract

**Background:**

Widely used rod rotation and translation techniques for idiopathic scoliosis (IS) are effective in correcting spinal coronal deformity. Bilateral apical vertebral derotation technique by vertebral column manipulation (VCM) and vertebral coplanar alignment (VCA) technique are two strategies for three-dimensional (3D) correction for IS. The purpose of this study is to compare the post-surgical results and technical features of the bilateral apical vertebral derotation technique by VCM against the VCA technique in patients with Lenke type 1 IS.

**Methods:**

Forty-eight patients with Lenke type 1 IS were enrolled in the present prospective clinical assay. They were divided into groups A (bilateral apical vertebral derotation technique by VCM, n=24) and B (VCA technique, n=24). Radiographic parameters measured before and after surgery included the Cobb angle, thoracic kyphosis, and apical vertebral rotation. Scoliosis Research Society (SRS)-22 scores were evaluated during the final follow-up. The differences in the demographics, surgical details, and radiographic measurements between the two groups were determined using a *T* test. The Mann–Whitney *U* test was used to evaluate the differences in the SRS-22 scores. A value of P<0.05 was considered statistically significant.

**Results:**

In the coronal plane, a significant difference was found in the correction rate of the major curve (group A: 84.8%, group B: 78.4%; P=0.045) and in the Cincinnati Correction Index between two groups (group A: 2.21, group B: 1.98; P=0.047). In the sagittal plane, no difference was found in the postoperative thoracic kyphosis between the two groups (P=0.328). In the transverse plane, no difference was found between the two groups in the correction rates of the rotation angle sagittal (P=0.298), rib hump (P=0.934), apical vertebral body-to-rib ratio (P=0.988), or apical rib spread difference (P=0.184). Patients underwent follow up for an average of 21.9 and 22.2 months in groups A and B, respectively. Results obtained at the final follow-up indicated no significant loss of correction. No differences were found in the SRS-22 scores between the two groups. No aortic or neurological complications were observed.

**Conclusions:**

The 3D deformity of the spine was effectively corrected using the bilateral apical vertebral derotation technique by VCM and the VCA technique, and encouraging post-surgical results were obtained for patients with Lenke type 1 IS. The two techniques were effective in allowing 3D correctional force that was applied in different ways.

## Background

Idiopathic scoliosis (IS) is a three-dimensional (3D) deformity of the spine. Interaction exists among the deformity in each plane, which determines the progress of scoliosis and the outcomes of surgery [1,2]. Correcting rotational deformity and maintaining the normal sagittal profile of the spine is more important than only correcting coronal deformity [3,4]. Since the introduction of Cotrel–Dubousset instrumentation in 1984, posterior correction techniques for IS have evolved from the Harrington system to pedicle screw instrumentation. Several comparative studies have suggested that pedicle screws provide better correction than Harrington rods [5-7]. Currently, popular pedicle screw correction techniques begin with rod rotation and translation techniques, which are effective for correcting coronal deformities [8,9].

The insufficient correction of rotation and sagittal deformities for IS has resulted in the development of new techniques for more effective 3D correction [10-12]. Bilateral apical vertebral derotation by vertebral column manipulation (VCM) was introduced by Chang and Lenke [13] using a “quadrilateral frame” to bilaterally simultaneously manipulate the spine, thereby effectively correcting 3D deformities present in IS. Vertebral coplanar alignment (VCA) was described by Vallespir [14] who utilized the coplanar nature of the *x*- and *z-*axes of the spine to correct translation and rotation in patients with scoliosis. Clinical studies have demonstrated the effectiveness of 3D correction for IS using VCA [14,15]. However, few reports to date have compared the bilateral apical vertebral derotation technique by VCM to the VCA technique as assessed by radiographic, functional, and outcome parameters, and technical features in patients with IS.

The effects of 3D correction, Scoliosis Research Society (SRS)-22 scores, and technical features of the bilateral apical vertebral derotation technique by VCM to the VCA technique for Lenke type 1 IS patients were compared.

## Methods

This study was approved by the institutional review board of the West China Hospital (Chendu, China). Written informed consent for participation in the study was obtained from each participant or, where participants are children, a parent, and written informed consent was also obtained for the publication of clinical images. Only one senior spine surgeon performed all surgeries. Data was collected by two independent spinal surgeons who were not involved in either the surgery or the post-surgical management of patients, and the mean was obtained. The patients were chosen according to the following criteria: aged between 10 to 21 years; Lenke type 1 IS with the major spinal curvature having a Cobb angle of 45° to 75° as confirmed independently by two experienced spinal surgeons according to the same standard (the Cobb method of measurement); and patients who underwent selective thoracic fusion. Patients were excluded if they had any observed neurological abnormality when examined clinically or by magnetic resonance imaging, if their main spinal curvature was toward the left, or if the pedicle of the apical vertebra was too small to place screw anchors adequately. From June 2010 to March 2011, forty-eight patients with Lenke type 1 IS were enrolled into the study that investigated two different spine curvature correction techniques. According to alternating group A and group B in the order of hospital admission of patients, twenty-four patients enrolled in group A underwent bilateral apical vertebral derotation by VCM. The rest of the patients were enrolled in group B and received VCA. Clinical demographic characteristics of patients in groups A and B are reported in Table 1. IS was classified according to Lenke et al. [16].

**Table 1:** Demographic data

	Group A*	Group B*
No. patients	24	24
Age at surgery (years ± SD)	14.3±2.4	15.1 ±3.2
Male/female	8/16	9/15
Risser sign at surgery(N ± SD)	3.5 ±1.7	3.6 ±1.3
Flexibility of major curve (% ± SD)	37.2±9.7	38.8±10.3
Lenke IS lumbar spine modifier, N (%)		
A	14 (58)	15(62)
B	6 (25)	5 (21)
C	4 (17)	4 (17)
Kyphosis (T5-T12 Cobb angle), N (%)		
Hypokyphosis (inferior to 10°)	4 (17)	4(17)
Normokyphosis (between 10° and 40°)	16 (66)	17(70)
Hyperkyphosis (superior to 40°)	4 (17)	3(13)

*Group A, bilateral apical vertebral derotation by VCM; Group B, VCA.

Surgical procedures for patients in groups A and B were identical, except for the correction technique (the bilateral apical vertebral derotation by VCM or the VCA, respectively). Fixation and fusion levels were in accordance with Lenke standards [16]. The Legacy screw-rod system (Medtronic, US) with rod diameter of 5.5 mm was employed; the diameters of the screws were 4.5, 5.5, and 6.5 mm and were used in the upper, middle, and lower thoracic vertebrae, respectively. The 6.5 mm screws were also used in the lumbar vertebrae.

The basic principle and procedure for bilateral apical vertebral derotation technique by VCM was used as described by Chang and Lenke [13]. A few slight technique modifications for selective thoracic fusion were made. Briefly, the pedicle screws were placed in every two to three segments on both sides within the fixation and fusion levels, and another multi-axial reduction screw was placed in the next vertebra of apical vertebra on the concave side. The VCM device was assembled in the three levels of apical and adjacent vertebra needed to fix it in place. In a flexible deformity, the two-level derotation device can be utilized effectively. After assembly, with ventral and medially-directed spinal implant forces performed using the vertical and convex derotator handles, a periapical derotational maneuver was assessed to quantify the degree of derotational corrective forces to be applied. With continued derotation and translational forces applied to the spine by the VCM device, the rod contoured in the sagittal plane only was engaged into the saddle of the pedicle screws on the concave side, and screws were braced with further distraction force. The VCM device was then retrieved. The rod on the convex side was inserted and appropriately compressed to improve further the correction.

The procedure for the VCA technique was performed as described by Vallespir [14]. Pedicle screws were placed in every vertebral segment on the convex side (some were removed after the correction by VCA for selective thoracic fusion) and in every two to three segments on the concave side. Slotted tubes were attached to the screws on the convex side. Two rigid bars were inserted through the uppermost part of the tubes. The upper bar held the original position, and the lower bar was progressively lowered toward the head of the screws. Then, spacers of varying lengths were placed between the tops of the tubes reconstructing normal kyphosis. For the patient with hyperkyphosis, a bandage was wound (the space was 20 mm to 30 mm) between the tops of the tube to reconstruct the normal kyphosis. After the correction was completed, a precontoured rod (only in the sagittal plane) was inserted and secured to each screw on the concave side with distraction force. VCA instrumentation and temporary screws were removed. The rod on the convex side was put in place, and compression force was used to correct further the deformity.

Then, two cross-links were used between two rods, and posterior grafting with allogeneic bone was performed. No patient underwent soft tissue release, osteotomy, and thoracoplasty.

**Figure 1:**
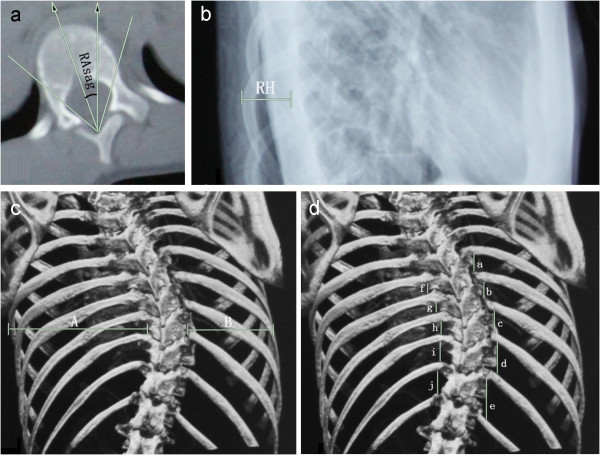
**Indices to assess the correction of apical rotation. a**) RAsag: the angle between the middle line of the apical body and the sagittal line by CT; **b**) RH: the distance between the right and left posterior rib humps in the lateral radiographs of apical vertebrae level; **c**) AVB-R: the ratio of linear measurements from lateral borders of apical vertebrae to chest wall in the anteroposterior radiograph (A/B); **d**) ARSD: the difference in the sums of the intercostal distances at the five periapical segments measured at the lateral transverse process by anteroposterior radiograph [(a+b+c+d+e) - (f+g+h+i+j)].

Radiographs of the entire spine and CT scans of the apical vertebra (performed in 39 of 48 patients: 20 in group A and 19 in group B) were used to compare the effects of 3D correction. Preoperative curve flexibility was determined on the preoperative supine-side bending films. The correction rate of the Cobb angles of the major curves and Cincinnati Correction Index (CCI) [17] were used to evaluate the correction effect in the coronal plane. The correction rate of Cobb angles was calculated as [(preoperative value-postoperative value)/preoperative value]×100%. CCI was calculated as correction rate of Cobb angles/preoperative curve flexibility. Another parameter was the translation of the apical vertebra as the distance from the perpendicular line drawn from the center of the S1 vertebral body to the center of the apical vertebral body or disk. The thoracic kyphosis (the Cobb angle from the T5 upper endplate to the T12 lower endplate on the lateral radiographs) was measured to assess the sagittal correction, and less than 10° was regarded as flatback deformity. The lumbar lordosis (the Cobb angle from the L1 upper endplate to the S1 upper endplate on the lateral radiographs) was also measured. Indices used to assess the correction of apical rotation included rotation angle sagittal (RAsag), rib hump (RH), apical vertebral body-to-rib ratio (AVB-R), and apical rib spread difference (ARSD) (Figure 1). The correction rate of these indices was calculated as [(preoperative value - postoperative value)/preoperative value] ×100%.

SRS-22 scores were assessed for every patient at the final follow-up visit to evaluate the function, pain, general self image, mental health, and satisfaction with treatment.

The statistical differences between the two groups were analyzed using SPSS18.0. The results were expressed as mean±standard deviation (SD). The differences in the demographics, surgical details, and radiographic measurements between the two groups were determined using *T* test. The Mann–Whitney *U* test was used for the valuation of the differences in the score of the SRS-22. A value of P<0.05 was considered statistically significant.

## Results

**Figure 2:**
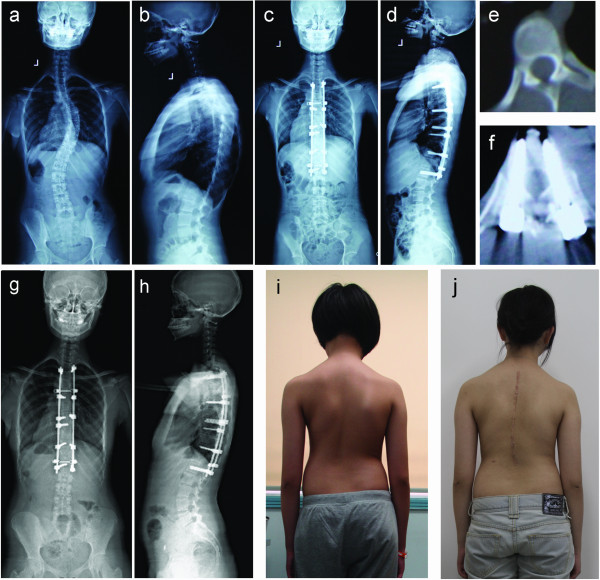
**Case 1.** A 13-year-old female patient with Lenke type 1BN IS was treated with bilateral apical vertebral derotation by VCM. **a**, **b**) Preoperative major curve Cobb angle (**a**) was 55.6°, and thoracic kyphosis (**b**) was 38.1°; **c**, **d**) Postoperative major curve Cobb angle (**c**) was 4.8°, and the thoracic kyphosis (**d**) was 24.2°; **e**, **f**) Preoperative (**e**) and postoperative (**f**) RAsag were 16.1° and 6.3°, respectively; **g**, **h**) Twenty-four months after surgery, radiographs show that the correction over the instrumented levels was maintained; **i**, **j**) Preoperative and postoperative clinical pictures demonstrating cosmetic changes.

**Figure 3:**
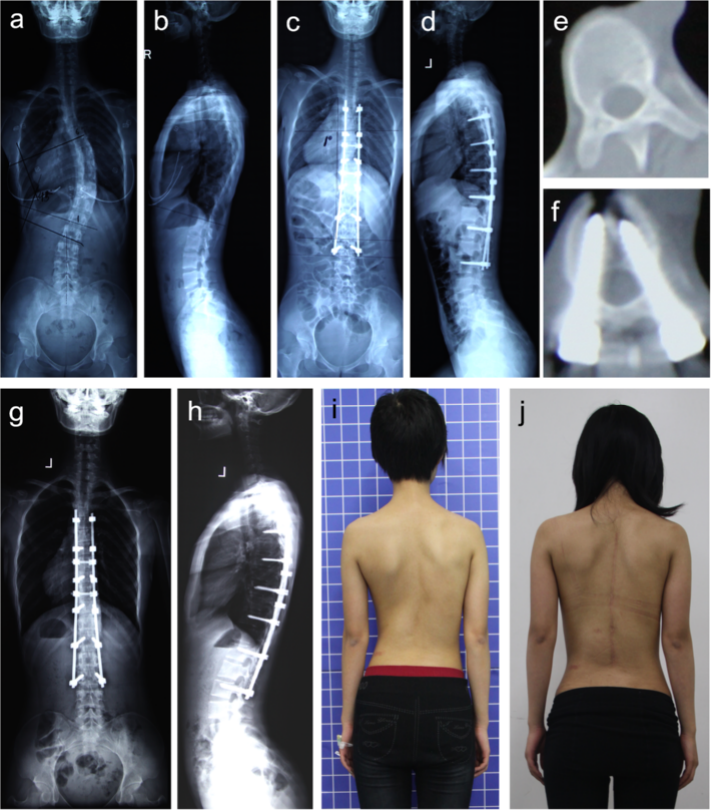
**Case 2.** A 20-year-old female patient with Lenke type 1AN IS was treated with VCA technique. **a**, **b**) Preoperative major curve Cobb angle (**a**) was 45.3°, and the kyphosis (**b**) was 14.2°; **c**, **d**) Postoperative major curve Cobb angle (**c**) was 4.1°, and the kyphosis (**d**) was 16.4°; **e**, **f**) Preoperative (**e**) and postoperative (**f**) RAsag were 17.8°and 7.6°, respectively; **g**, **h**) Twenty-five months after surgery, radiographs show that the correction over the instrumented levels was maintained; **i**–**j**) Preoperative clinical and final follow-up pictures demonstrating cosmetic changes.

No statistical differences between groups A and B in terms of age, Risser sign at surgery, and flexibility of major curve were found (P>0.05) (Table 1). No statistical differences in the preoperative Cobb angle, thoracic kyphosis, RAsag, RH, AVB-R, and ARSD were noted (P>0.05). Surgery was successful for all patients, without aortic or neurological complications. The pedicle screws of the apical vertebra were successfully placed in all cases. The range of levels fused, implant density, and other surgical information are listed in Table 2. No differences in these surgical parameters between the two groups were found (P>0.05). Patients underwent follow up for an average of 21.9 months (range 18 months to 26 months) in group A, and an average of 22.2 months (range 18 months to 26 months) in group B (Figures 2 and 3).

**Table 2:** Surgery details

	Group A*	Group B*
Instrumented level (N ± SD)	12.1±1.5	11.8±1.8
Implant density (% ± SD)	55.7±5.6	53.8±8.3
Operation time (min ± SD)	157±54	168±65
Intraoperative blood loss (ml ± SD)	664±269	687±248

*Group A, bilateral apical vertebral derotation by VCM; Group B, VCA.

For both groups, the preoperative and postoperative Cobb angle of the major curve and translation of the apical vertebra are provided in Table 3. The difference in the correction rate of major curve was statistically significant between groups A and B (84.8% and 78.4%, respectively, P=0.045). Statistical difference in CCI between the two groups was found (2.21 in group A and 1.98 in group B, P=0.047). During the final follow-up visit, the amount of correction loss of the major curve Cobb angle was not significantly different in groups A and B compared with the post-operative measurement. The difference in translation of the apical vertebra was not statistically significant before and after surgery when the two groups were compared (P>0.05).

**Table 3:** Comparison of the effects of three-dimensional correction

	Group A^*^			Group B^*^		
	**Preoperative**	**Postoperative**	** Final**	**Preoperative**	**Postoperative**	** Final**
			**Follow-up**			**Follow-up**
Major curve Cobb angle (degree ± SD)	58.9±9.6	8.8±4.7**	9.7±5.6	62.3±11.9	13.4±7.1**	14.5±6.5
Apical translation (mm ± SD)	55.7±13.2	13.8±8.4**	15.1±7.3	49.3±13.9	12.5±10.9**	14.8±9.2
RAsag (degree ± SD)	17.5±5.4	6.6±3.1**	7.0±3.7	19.8±6.2	8.3±3.3**	9.0±2.9
RH (mm ± SD)	28.6±9.3	13.1±5.2**	14.2±6.5	24.1±10.9	11.2±7.2**	12.1±8.4
AVB-R (ratio ± SD)	2.0±0.4	1.4±0.3**	1.4±0.6	2.3±0.3	1.6±0.5**	1.7±0.4
ARSD (mm ± SD)	48.3±15.7	9.4±6.1**	10.7±8.0	45.7±16.3	11.2±7.9**	12.0±8.6
Thoracic kyphosis angle (degree ± SD)	29.7±19.8	20.1±9.3**	22.7±11.7	22.4±15.3	19.9±8.9	21.5±13.0
Lumbar lordosis angle (degree ± SD)	53.4±9.5	44.2±7.3**	48.3±9.1	44.9±15.4	48.3±8.6	49.5±10.0
Thoracic kyphosis angle in flat back patients (degree ± SD)	7.1±1.4	11.3±2.0	12.0±2.6	5.2±3.1	13.4±1.5**	13.8±2.3

^*^Group A, bilateral apical vertebral derotation by VCM; Group B, VCA.

**P<0.05 indicates statistically significant difference in the postoperative value compared with preoperative value.

No difference in postoperative thoracic kyphosis was noted between the two groups (P=0.328). In group A, the postoperative thoracic kyphosis was less than the preoperative thoracic kyphosis (P=0.024). The flatback deformity in four patients was corrected after surgery, without statistically significant difference in thoracic kyphosis angle compared with the preoperative value (P=0.258). In group B, the postoperative thoracic kyphosis was not different from the preoperative thoracic kyphosis (P=0.746). However, in the four patients who had flatback deformity before surgery, the thoracic kyphosis angle was statistically different before and after surgery (P=0.033). The difference in preoperative and postoperative lumbar lordosis between the two groups was not statistically significant (P>0.05). The thoracic kyphosis and lumbar lordosis corrections were well maintained during follow-up in both groups (Table 3).

Preoperative and postoperative RAsag, RH, AVB-R, and ARSD in both groups are provided in Table 3. No significant difference was found between the two groups in the correction rate of RAsag (61.5% and 56.7% in groups A and B, respectively; P=0.298), RH (55.3% and 54.6% in groups A and B, respectively; P=0.934), AVB-R (28.0% and 27.9% in groups A and B, respectively; P=0.988), and ARSD (83.6% and 72.1% in groups A and B, respectively; P=0.184). During the final follow-up visit, the amount of correction loss of RAsag, RH, AVB-R, and ARSD was not significantly different in both groups compared with the post-operative measurements.

During the final follow-up visit, SRS-22 scores were used to evaluate each patient. The mean SRS-22 questionnaire scores for function, pain, general self-image, mental health, and satisfaction with treatment for patients were 3.8, 4.4, 4.6, 4.5, and 4.3, respectively in group A, and 3.7, 4.5, 4.3, 4.3, and 4.4, respectively, in group B. No difference was noted between groups A and B in any of these domains (P>0.05) (Table 4).

**Table 4:** Comparison of the Scoliosis Research Society (SRS)-22 scores

	Group A*	Group B*
Function/activity (scores ± SD)	3.8±1.2	3.7±1.3
Pain (scores ± SD)	4.4±1.7	4.5±1.8
Self-image/appearance (scores ± SD)	4.6±2.0	4.3±1.7
Mental health (scores ± SD)	4.5±.0.8	4.3±1.1
Satisfaction with management (scores ± SD)	4.3±1.5	4.4±1.9
Total (scores ± SD)	94.6±6.7	92.9±8.7

*Group A, bilateral apical vertebral derotation by VCM; Group B, VCA.

## Discussions

To achieve 3D correction for IS, many strategies have been used, from the development of the Harrington system in the late 1950s to pedicle screw instruction in the 1990s, which provided better correction and stability for patients than did the Harrington system [4-7]. The rod rotation and translation technique with pedicle screw instruction is the most commonly used correction technique for IS, which is effective for coronal deformity [8,9]. Many new correction techniques have been developed to correct effectively 3D deformities, such as simultaneous derotation using two rods, simultaneous translation using two rods, direct vertebral rotation, bilateral apical vertebral derotation by VCM, and VCA [3,10-14].

The principle of bilateral apical vertebral derotation technique by VCM is that IS can be corrected in three dimensions by the manipulation of the VCM quadrilateral frame configured in the apical vertebral area. Translational forces could be applied to the device for medialization of the apex of the deformity in the coronal plane toward the concave correcting rod. Manipulation of the convex handles provides apical vertebral derotation, and corresponding manipulation of the vertical handles may aid in the correction of the deformity in the sagittal plane [13].

VCA is based on the theory that in normal spine, the *x-* and *z-*axes of the different vertebrae aligned in a single plane, and rotation and translation of these vertebrae only exist in the sagittal plane. However, a loss of the normal coplanar alignment exists in scoliosis. The principle of VCA is based on both returning the normally coplanar axes (*x* and *z*) into a single plane and returning the *x-*axis to its normal posterior divergence in the sagittal plane [14].

In this study, IS patients with the major curve Cobb angle of 45° to 75° were enrolled to investigate the derotational correction and the improvement of body image. However, for IS patients with the major curve Cobb angle of more than 75°, the security and effective correction of the coronal plane of the spine were the most important considerations. If the pedicle diameter of the apical vertebra was too small to place screw anchors adequately, VCM technique would not be used for these IS patients. Otherwise, there would be the possibility of the pedicle burst during correction, because of the concentration of correction force in the apical vertebra area by VCM.

Coronal correction was studied earlier than the two other dimensions for IS, and as previously reported, the average coronal correction is approximately 70% [5,7,8,10-12]. Using VCA for Lenke type 1 IS, Vallespir [14] reported that the coronal correction rate was 73.3%, and Yong Qiu [15] described a coronal correction rate of 71.8%. In our study, although a difference was found in the correction rate of the major curve between the bilateral apical vertebral derotation technique by VCM (84.8%) compared with the VCA technique (78.4%), the coronal correction rates in both groups were satisfactory and higher than the average level reported. This result may be caused by the absence of large values of preoperative Cobb angle (>75°) and the absence of small values for flexibility (<30%) [18,19] in our patients.

Restoring the sagittal balance of the spine is one of the most important goals in IS surgery. Controversies about the role of the pedicle screw instruction in the restoration of thoracic kyphosis have been discussed. Kim [5] observed a decrease of 14° in the thoracic kyphosis. Lowenstein [7] reported a decrease of 10° in kyphosis. However, Suk [8] reported an improvement in kyphosis, and Jean-Luc [10,12] achieved a gain of 23° in kyphosis with simultaneous translation using the two-rod technique. Results from this study showed a decrease in the mean kyphosis in the bilateral apical vertebral derotation by VCM (from preoperative 29.7° to postoperative 20.1°) but not in the patients who underwent VCA (from 22.4° to 19.9°). However, in our study, grouping of the kyphosis angle around normality in both groups was found, with a decrease in the higher values and an increase in the lower ones. This finding may be explained by the theory that changes in thoracic kyphosis on fulcrum bending caused by natural coupling of deformities are directed toward self-normalization [2]. Results from the present study also showed a significant improvement in kyphosis in the VCA group for the flatback deformity in patients (preoperative 5.2° to postoperative 13.4°). This improvement might be caused by VCA, which can correct sagittal plane deformities in every segment compared with VCM, mostly in the apical vertebral area.

Measurements of vertebral rotation are made using the RAsag on CT as described by Aaro and Dahlborn [20]. The RH, AVB-R and ARSD values were obtained through radiographs. The average RAsag correction rate using rod rotational and translational techniques was approximately 10% [21]. Lee [3] reported 42.5% rotational correction by direct vertebral rotation. Vallespir [14] measured the rotational correction obtained by the VCA, and reported a correction of 56%. Steib [22] achieved vertebral rotation correction in approximately 60% of patients by in situ contouring technique using 3D reconstruction. Other studies have shown that the correction rate of RH ranged from 51% to 65%, and the correction rate of AVB-R ranged from 29% to 54% [9,14]. Results of our study have shown that the correction rate of RAsag in both groups (61.5% in the bilateral apical vertebral derotation by VCM group and 56.7% in the VCA group) was higher than that of derotation techniques and direct vertebral rotation technique [3,21], but similar with the correction rate of VCA reported by Vallespir [14]. The correction rate of RH and AVB-R in our study was similar to those of other reports [9,14]. As mentioned above, the current correction rate of RAsag was approximately 60%, which is consistent with results observed in this study. Thus, we hypothesized that the structural change in the spine limits the amount of correction possible in vertebral rotation in patients with IS, not the correction technique. This hypothesis is supported by Beuerlein [23] who found that structural changes in the spine of a patient with scoliosis, including disc and vertebral wedging, develop as the spinal curves “matures”. In addition, the vertebrae could be “fixed” in axial rotation, but that they are unresponsive to attempts to improve the axial deformity.

The advantage of bilateral apical vertebral derotation technique by VCM is that the VCM assembly allows for the maximum degree of translation and derotation force to be safely applied to the periapical region of the spine. Thus, we obtained a better correction rate of the major curve in group A than in group B which was treated by VCA. Cheng [24] performed a biomechanical analysis of the derotation of the thoracic spine using pedicle screws and found that quadrangularly linked pedicle screws allow for significantly greater torque (with failure at 42.5 Nm±14.5 Nm) compared with a single pedicle screw, bilaterally linked screw, and unilaterally linked pedicle screw constructs. Another advantage is that VCM quadrilateral frame could reduce the possibility that the concave screw may need to be removed because of its proximity to the aorta after the correction by direct vertebral rotation technique [25]. The limitation of this technique is that the success of manipulation of the VCM frame depends on the experience of the surgeon, because too much force could lead to fractures in the periapical region of the vertebrae. However, if the force applied to the VCM frame is insufficient, the correction will not be as successful.

The advantages of VCA are as follows: (1) pedicles at the convex side could provide more powerful force than at the concave side in the rod rotation and translation technique, and correction on the convex side helps prevent spinal injury during the surgery; (2) the corrected force acts on each segment, and the correction is achieved in a step-by-step process, which could effectively reduce the risk of screw extraction and neurological injury as well as restore normal sagittal offset; and (3) manipulation of the VCA is simple, requiring manipulation of only the lower rigid bar downward along the slots of the tubes.

Moreover, we did not place pedicle screws at every level in the fixation-fusion segment in both VCM and VCA groups. So there was the difference between our surgical technique of VCM and the original technique, and in VCA group some pedicle screws also had to be removed after VCA. However, by this way the satisfactory correction effect of IS with less cost could be offered to our patients. Whether the correction effect of our method of placing screws is equal to the original technique in VCM and VCA, need to be further study by biomechanical analysis and clinical assay.

The sample size in this study is relatively small, and a larger study is needed to assess further the effects of bilateral apical vertebral derotation technique by VCM and VCA on spinal correction in patients with Lenke type 1 IS. Moreover, a longer follow up time is needed to determine how well the spinal curvature corrections are maintained in these patients.

## Conclusions

For patients with Lenke type 1 IS, the 3D deformity of the spine was effectively corrected, and encouraging post-surgical results were obtained using both the bilateral apical vertebral derotation technique by VCM and the VCA technique. These two techniques provided effective 3D correction for IS using forces in different ways.

## Abbreviations

IS: Idiopathic scoliosis; VCM: Vertebral column manipulation; VCA: Vertebral coplanar alignment; 3D: three-dimensional; CCI: Cincinnati Correction Index; SRS-22 scores: Scoliosis Research Society-22 scores; RAsag: Angle sagittal; RH: Rib hump; AVB-R: Apical vertebral body-to-rib ratio; ARSD: Apical rib spread difference.

## Competing interest

The authors have declared that no conflict of interest exists.

## Authors’ contributions

SL carried out the study design and literature research, participated in clinical studies, and drafted the manuscript. SY carried out the study concepts and clinical studies. LL participated in the study design, clinical studies, and manuscript review. ZC and ZZ performed data acquisition and analysis. AY performed the statistical analysis and helped draft the manuscript. All authors read and approved the final manuscript.

## Pre-publication history

The pre-publication history for this paper can be accessed here:

http://www.biomedcentral.com/1471-2474/14/175/prepub
